# Correction and removal of expression of concern: Microgel-stabilised non-aqueous emulsions

**DOI:** 10.1039/d6ra90114j

**Published:** 2026-09-04

**Authors:** Amro K. F. Dyab, Ayman M. Atta

**Affiliations:** a Chemistry Department, Faculty of Science, Minia University Minia 61519 Egypt amr.diab@mu.edu.eg; b Department of Chemistry, College of Science, King Saud University P.O. Box 2455 Riyadh 11451 Saudi Arabia

## Abstract

Correction and removal of expression of concern for ‘Microgel-stabilised non-aqueous emulsions’ by Amro K. F. Dyab *et al.*, *RSC Adv.*, 2013, **3**, 25662–25665, https://doi.org/10.1039/C3RA45263H.

The authors regret unattributed data overlap between their article and ref. 1. Fig. 1 was re-used from ref. 1 without being correctly attributed and without permission from the publisher. In addition, there was an error in the caption.

The authors have now received permission to reuse the image and the corrected caption is below.

**Fig. 1** TEM image for 98 : 2 mol% (NIPAM/AMPS) microgels. Reproduced from Ayman Atta *et al.*, *Polym. Adv. Technol.*, **24**(11), 986–996, with permission from Wiley.

This notice supersedes the information provided in the expression of concern related to this article.

The Royal Society of Chemistry apologises for these errors and any consequent inconvenience to authors and readers.
